# Robotic-Assisted Colon Cancer Surgery: Faster Recovery and Less Pain Compared to Laparoscopy in a Retrospective Propensity-Matched Study

**DOI:** 10.3390/cancers17020243

**Published:** 2025-01-13

**Authors:** Chun-Yu Lin, Yi-Chun Liu, Chou-Chen Chen, Ming-Cheng Chen, Teng-Yi Chiu, Yi-Lin Huang, Shih-Wei Chiang, Chang-Lin Lin, Ying-Jing Chen, Chen-Yan Lin, Feng-Fan Chiang

**Affiliations:** 1 Institute of Clinical Medicine, National Yang Ming Chiao Tung University, Taipei 112211, Taiwan; classicpiano2003@gmail.com (C.-Y.L.);; 2 Division of Colorectal Surgery, Department of Surgery, Taichung Veterans General Hospital, Taichung 40705, Taiwan; 3 School of Medicine, National Defense Medical Center, Taipei 11466, Taiwan; 4 School of Medicine, National Yang Ming Chiao Tung University, Taipei 112211, Taiwan; 5 Department of Radiation Oncology, Taichung Veterans General Hospital, Taichung 40705, Taiwan; 6 Department of Post-Baccalaureate Medicine, College of Medicine, National Chung Hsing University, Taichung 402202, Taiwan; 7 College of Humanities and Social Sciences, Providence University, Taichung 433303, Taiwan

**Keywords:** robotic surgery, colorectal cancer, colon cancer, minimally invasive surgery, laparoscopy

## Abstract

**Background and Objective**: Colorectal cancer (CRC) is the third most common cancer worldwide, with colon cancer accounting for approximately 60% of all CRC cases. Surgery remains the primary and most effective treatment. Robotic-assisted surgery (RAS) has emerged as a promising approach for colon cancer resection. This retrospective study compares RAS and laparoscopic-assisted surgery (LSS) for stage I–III colon cancer resections at a single medical center in East Asia. **Methods**: Between 1 January 2018, and 29 February 2024, patients undergoing colectomy were classified into right-side and left-side colectomies. Propensity score matching was conducted based on age group, gender, ASA score, and BMI to ensure comparability between groups. After matching, there were 50 RAS and 200 LSS cases for right colectomy (RC), and 129 RAS and 258 LSS cases for left colectomy (LC). Perioperative outcomes were compared between the two surgical approaches. The primary outcomes were recovery milestones, while secondary outcomes included complications and postoperative pain scores. **Results**: RAS demonstrated faster recovery milestones compared to LSS (hospital stay: 6.5 vs. 10.2 days, *p* = 0.005 for RC; 5.5 vs. 8.2 days, *p* < 0.001 for LC). RAS also resulted in lower rates of ileus (14% vs. 26%, *p* = 0.064 for RC; 6.2% vs. 15.9%, *p* = 0.007 for LC) and higher lymph node yields (31.4 vs. 26.8, *p* = 0.028 for RC; 25.8 vs. 23.9, *p* = 0.066 for LC). Major complication rates showed no significant difference between RAS and LSS (4.0% vs. 7.0%, *p* = 0.746 for RC; 4.7% vs. 3.1%, *p* = 0.563 for LC). Patients in the RAS group experienced earlier diuretic phases and reported significantly lower postoperative pain scores (3.0 vs. 4.1, *p* = 0.011 for RC; 2.9 vs. 4.1, *p* < 0.001 for LC). **Conclusions**: Robotic-assisted surgery is associated with faster recovery, lower rates of ileus (LC), higher lymph node yield (RC) and reduced postoperative pain compared to laparoscopic-assisted surgery for colon cancer resection.

## Simple Summary

Colorectal cancer is the third most common cancer worldwide, with colon cancer accounting for approximately 60% of all cases. Robotic-assisted surgery has emerged as a promising minimally invasive technique for resection. This study evaluates and compares the outcomes of robotic-assisted surgery and laparoscopic-assisted surgery for stage I to III colon cancer, using data collected between 2018 and 2024. By analyzing matched patient groups, the findings demonstrate that robotic-assisted surgery leads to faster recovery, fewer cases of bowel obstruction in left-sided colectomies, greater lymph node retrieval in right-sided colectomies, and reduced postoperative pain compared to laparoscopic-assisted surgery. These results underscore the potential of robotic-assisted surgery to enhance recovery and improve surgical precision. The study provides valuable evidence that may influence surgical practices and promote the broader adoption of robotic technology, particularly in the treatment of colon cancer.

## 1. Introduction

Colorectal cancer is one of the most common cancers worldwide. It ranks third in terms of incidence and second in terms of cancer-related deaths globally. The global incidence of CRC is approximately 1.9 million new cases per year (as of 2020). The highest incidence rates are found in developed countries, including Europe, North America, and Australia. However, its incidence is increasing in some Asian, South American, and Eastern European countries due to Westernized lifestyle changes. The treatment approach for colorectal cancer primarily depends on the stage of the disease. Stages I to III are considered localized diseases. Among various treatment options, radical resection remains the most effective method for colorectal cancer. Stage III cancer, due to lymph node involvement, carries a higher risk of spreading to other organs, making six months of adjuvant chemotherapy (12 cycles) recommended after surgery. Stage IV cancer, having metastasized to other organs, requires systemic therapy along with surgical resection to control the spread of cancer cells. The treatment is more arduous, and even so, the five-year survival rate for stage IV remains below 20%. Therefore, early detection is key to cancer treatment. Colon cancer accounts for approximately 60% of all colorectal cancer cases. Surgical resection at an early stage is the most effective therapy.

The development of minimally invasive surgery significantly advanced after the evolution of optical lenses in the 1980s. In 1985, Dr. Erich Mühe performed the first laparoscopic cholecystectomy [1]. In 1991, Jacobs M. reported the first laparoscopic colon surgery [2], confirming the feasibility of laparoscopic procedures in abdominal surgery. Studies have demonstrated that laparoscopic surgery offers a faster recovery compared with traditional open surgery without compromising oncological outcomes [3,4]. As more surgeons have gained experience with laparoscopic techniques, these procedures have become increasingly standardized. The development of advanced laparoscopic instruments and techniques, such as staplers, energy devices, and improved visualization systems, have further enhanced the safety and efficacy of laparoscopic colorectal surgery, with open surgery rates declining annually [5].

Robotic-assisted laparoscopic surgery (RAS) offers enhanced dexterity, precision, and visualization, especially in complex surgeries. Its applications were initially emphasized in urology. The first RAS colectomies were reported in 2002, by Weber et al. for benign disease [6] and by Hashizume et al. for malignant disease [7], but the applications remained limited by the instruments’ condition. After 2015, since the improvement of the platform and instruments, RAS became widespread and popular, especially in the gastrointestinal surgery field, which includes colorectal surgery [8,9]. However, RAS has limitations, such as longer operation times, higher costs, and the learning curve [10,11]. Colon cancer resection is regarded as a relatively simple procedure in this field, and the indication for RAS is weaker. The effect of RAS in colon cancer resection compared with conventional laparoscopic surgery (LSS) is still debated. Considering these factors, this study analyzes the perioperative outcomes between RAS and LSS in colon cancer resection in a single medical center in East Asia.

## 2. Material and Methods

### 2.1. Dataset

This study retrospectively collected minimally invasive colon cancer resection surgery patients from 1 January 2018 to 29 February 2024 in a single institute in East Asia. The inclusion criteria were as follows: Patients with a diagnosis of colon cancer, clinical stages I–III, with an American Society of Anesthesiologists (ASA) score of three or below who underwent elective and curative colon resection involving either robotic-assisted or laparoscopic surgery. The exclusion criteria were as follows: Any patient with a tumor located in their rectal segment was excluded. The definition of a tumor located in the rectal segment [12] was a tumor observed within 15 cm from the anal verge, as seen during a colonoscopy, a tumor identified below the S3 level in images, or a tumor shown in specimen photographs as being located below the point where taeniae coli disappear, which is defined as the upper rectum segment above the peritoneal reflection. Other exclusion criteria included benign colon disease or malignancy from another organ; metastatic colon cancer; open surgery; emergent surgery; combination with another resection (liver, bladder, ureter, or uterus); and cases within the learning curve for the robotic system of each surgeon (the first twenty cases) and for laparoscopic surgery (sixty cases, including one specialty training year and the first two years of proctologist specialty). Cases with unclear medical records were also excluded. Ethical approval was obtained (IRB number: CE24324B), and this study was registered at ClinicalTrials.gov (NCT05210647).

### 2.2. Analysis Content

The following contents were analyzed.

Preoperative factors: Patient age, gender, body mass index (BMI), ASA anesthetic risk, tumor location, clinical stage, bowel preparation, patient-controlled analgesia (PCA) insertion, and enhanced recovery after surgery (ERAS) [13,14] protocol applications.

Intraoperative outcomes: Surgical procedure, surgery time, blood loss, blood transfusion events, placement of drainage tube, stoma creation, rate of conversion to open surgery, pathological staging, a positive pathological circumferential resection margin (CRM) (pCRM+) [15], tumor edge-to-colon cut end distal margin, and number of lymph nodes (LNs) harvested.

Postoperative recovery: Time to first drink of water, liquid diet intake, defecation, and time to urinary catheter removal and intravenous therapy (IV) removal. Additionally, the length of hospital stay was recorded.

The textbook outcome (TO) [16,17] is a novel surgical quality assessment tool that combines structure, process, and surgical outcome. We defined the TO as a hospital stay of less than 5 days (75th percentile), along with the absence of any 30-day complications, need for readmission, or mortality, while considering the National Health Insurance policy, where patients are allowed to stay extra days according to their willingness. The IV discontinuation (DC) textbook outcome was recalculated based on IV removal, indicating readiness for discharge.

Postoperative complications within 30 days were classified using Clavien–Dindo scores: grade I minor, where the patient requires symptomatic medication, IV support, bedside wound dressing, physical therapy, or nasogastric tube insertion; grade II minor, where the patient requires upgraded antibiotics, blood transfusion or total parenteral nutrition (TPN) support; grade III, where the patient requires radiologic or surgical intervention; grade IV major, where the patient experiences single or multiple organ failure and requires intensive care unit (ICU) treatment; and grade V major, where the patient died due to surgical factors within 30 days postoperatively. Common complications included ileus, wound infection, pneumonia, cardiovascular events, cardiopulmonary events, anastomotic leakage, and chylous ascites, all of which were separately recorded. Readmission to the hospital within 30 days and the reasons why were also analyzed [18].

The most commonly seen complication events were recorded and defined as follows. 1. Cardiovascular events: Any incident related to the heart or blood vessels occurring after surgery, which included myocardial infarction, non-ST-elevation myocardial infarction (NSTEMI), arrhythmia, deep vein thrombosis, or other cardiovascular disturbances. 2. Respiratory events: An infection or inflammation within the lungs that induced fever, chills, or difficulty in breathing, requiring the patient to be supported with an oxygen supplement or ventilation machine. 3. Ileus: The cessation of a normal bowel movement after surgery, which induced abdominal distension, nausea, and/or vomiting while also creating the inability to pass gas or stool. Any evidence of such complications was proved via a physical exam or an abdominal kidney, ureter, or bladder radiography (KUB) exam with either a dilated bowel loop (small bowel > 3 cm with a step-ladder appearance; peripheral colon > 6 cm; cecum > 9 cm) or air–fluid level. 4. Wound infection: An infection that occurred at the site of the surgical incision, with signs including redness, swelling, or pus discharge. 5. Anastomosis leakage: The leakage of fluids, such as bowel contents or bile juice from an anastomosis, as proven via a physical exam, images, and laboratory data. 6. Chyle: The accumulation of lymphatic fluid in the abdominal cavity, typically proved via laboratory data involving the enrichment of triglycerides or via images. 7. Urinary complications: An infection of the urinary tract system related to urine retention and subsequent fever, proved via laboratory data [19,20].

### 2.3. Main Outcomes and Data Management

The data were analyzed to compare the differences in recovery conditions and surgical outcomes between robotic-assisted surgery (RAS) and conventional laparoscopic surgery (LSS) in routine curative colon cancer resections. Propensity score matching was performed based on age, gender, lesion site, and ASA score due to differences in patient characteristics between the RAS and LSS groups.

Primary outcomes: Milestones of recovery.

Secondary outcomes: Detailed descriptions of the recovery process and pain levels. The patient’s overall fluid balance was used as an objective indicator of recovery status. Fluid status was calculated based on three components: intravenous fluids, urine output, and gastrointestinal intake, all of which were monitored to assesszs postoperative conditions during hospitalization. The diuretic phase, where the patient’s urine output exceeded the volume of intravenous fluids without the use of diuretics, was a strong indicator of recovery from surgical stress. Increased food intake signified the recovery of gastrointestinal function. Pain scores were also calculated as an indicator of quality of life. Pain scores were recorded at the highest values on each postoperation day according to the EHIS system.

### 2.4. Sample Power

A power analysis was conducted to detect a meaningful difference in the recovery time between the two groups, estimated at 5 days for the robotic-assisted surgery (RAS) group and 8 days for the conventional laparoscopic surgery (LSS) group. With a desired statistical power of 0.8, a total of 66 cases were required in each group. However, at least 200 cases in RAS and 600 cases in LSS were necessary to account for potential exclusions and ensure robust matching to ensure a sufficient sample size for analyzing sidedness and performing propensity score matching (PSM).

### 2.5. Statistical Methods

The quantitative data are expressed as means ± SD. One-way analysis of variance (ANOVA) with least significant difference (LSD) multiple comparisons was used to analyze the quantitative differences between the two groups. The groups were compared using *t*-tests, Mann–Whitney U tests, chi-square tests, and Fisher’s exact tests as appropriate. The statistical analyses were performed with the IBM SPSS 20.0 software (SPSS Inc., Chicago, IL, USA). Significance was set at *p* < 0.05.

## 3. Results

### 3.1. Patient Recruitment

**Figure 1:**
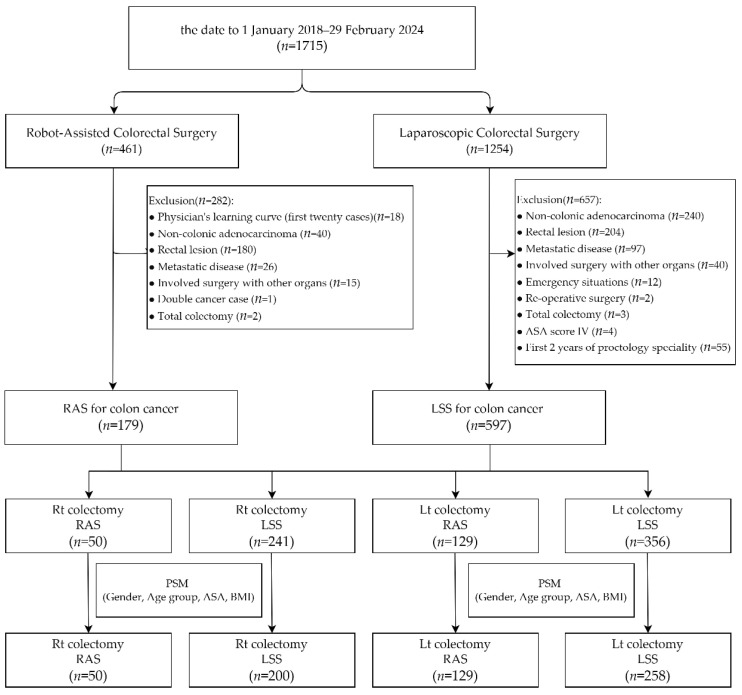
Depiction of patient selection and propensity score matching for this study.

From 1 January 2018 to 29 February 2024, a total of 1715 minimally invasive colorectal surgery patients were enrolled, with 461 of the surgeries having used robotic assistance and 1254 having been laparoscopic. Amongst the 461 robotic cases, the following were excluded: 180 cases that had a lesion located in the rectal segment, 40 that were diagnosed as non-colonic adenocarcinoma, 26 that were diagnosed as metastatic disease, 18 that were within the robotic system learning curve (the first 20 cases) of each surgeon, 15 that had undergone surgery involving other organs, 2 total colectomy cases, and 1 double cancer case. Amongst the 1254 cases that underwent laparoscopic surgery, the following were excluded: 240 cases involving non-colonic adenocarcinoma, 204 that were a lesion located in the rectal segment, 97 that were diagnosed as metastatic disease, 40 that involved surgery with other organs, 12 that were emergency situations, 4 that were classified as ASA score IV, 3 that were undergoing total colectomy, 2 that involved re-operative surgery, and 55 cases performed within the first two years of each proctologist’s career (the first 60 cases for each surgeon). A total of 179 cases of robotic-assisted surgery performed by two surgeons and 597 cases of laparoscopic surgery performed by fourteen surgeons for colon cancer were included. We divided the patients into two groups based on the type of surgery: right colectomy (RC) and left colectomy (LC). The RC group included 50 patients who underwent robotic-assisted surgery (RAS) and 241 patients who underwent laparoscopic surgery (LSS), while the LC group included 129 RAS patients and 356 LSS patients. After propensity score matching for gender, age group, ASA score, and BMI, the RC group consisted of 50 RAS patients and 200 LSS patients, and the LC group consisted of 129 RAS patients and 258 LSS patients (Figure 1).

### 3.2. Patient Characteristics

The composition of patients was generally similar for both right and left colectomies; however, a higher proportion of RAS patients participated in the ERAS program. At our institution, ERAS and RAS were introduced around the same time (May 2019 and January 2020, respectively), leading to a significantly higher rate of ERAS participation among RAS patients (RC: 82.0% vs. 8.0%, *p* < 0.001; LC: 94.6% vs. 15.1%, *p* < 0.001). The ERAS program was divided into three parts. Preoperative preparation: This included nutritional education, correction of anemia, and preoperative oral intake two hours before surgery. Intraoperative management: This focused on anesthesia-related factors and the avoidance of drainage tubes. Postoperative care: This emphasized early feeding and the discontinuation of intravenous fluids once bowel function was restored. Patient-controlled analgesia (PCA) was recommended to facilitate early ambulation, resulting in higher PCA usage rates in the RAS group (RC: 86.0% vs. 30.0%, *p* < 0.001; LC: 93.0% vs. 35.7%, *p* < 0.001). Before the implementation of ERAS, most physicians waited for patients to pass gas before allowing them to start drinking water. After drinking water, the subsequent treatment was similar to the ERAS protocol: patients would begin eating once gastrointestinal motility was confirmed, and intravenous fluids were gradually reduced as gastrointestinal function recovered.

Regarding surgeons, as RAS has only recently been introduced and has higher costs, it was initially performed by more senior surgeons during its early adoption (RC: 82% with >11 years of experience and 18% with 6–10 years; LC: 89.1% with >11 years and 10.9% with 6–10 years). Two surgeons performed on cases meeting the inclusion criteria during the study period. However, there has been a recent trend of RAS being adopted by younger, less senior surgeons.

Laparoscopic surgery has been widely adopted for many years, with extensive exposure starting during specialist training. As a result, a total of 14 surgeons performed laparoscopic procedures, all of whom had at least 3 years of experience as board-certified colorectal surgeons (RC: 53.5% with >11 years, 17.5% with 6–10 years, and 29.0% with 3–5 years; LC: 58.1% with >11 years, 15.5% with 6–10 years, and 26.4% with 3–5 years) (Table 1 and Table 2).

### 3.3. Intraoperative Outcomes and Pathology Features After Propensity Score Matching

In the matched data, RAS had a longer operative time compared with LSS (310.5 (271.5–352.0) vs. 190.5 (144.0–242.5), *p* < 0.001 in RC; 264.0 (229.0–309.0) vs. 187.0 (149.0–249.0), *p* < 0.001). The other outcomes were generally similar, although RAS demonstrated a higher number of lymph nodes harvested (31.4 ± 13.7 vs. 26.8 ± 10.6, *p* = 0.028 in RC; 25.8 ± 10.7 vs. 23.9 ± 9.2, *p* = 0.066 in LC). The conversion rate to open surgery was lower in RAS, though not statistically significant (0.0% vs. 3.0%, *p* = 0.603 in RC; 0.8% vs. 3.1%, *p* = 0.282 in LC) (Table 3). The reasons for stoma creation in two RAS cases and four LSS cases included three patients with underlying conditions, such as uremia and a history of coronary artery disease, one patient with partial colonic obstruction, and one patient requiring neoadjuvant radiotherapy for locally advanced descending colon cancer.

### 3.4. Postoperative Recovery and Complications

In the matched data, RAS patients initiated postoperative oral intake more aggressively than LSS patients, with earlier times for drinking water (0.7 vs. 2.4 days, *p* < 0.001 in RC; 0.5 vs. 2.0 days, *p* < 0.001 in LC) and liquid food consumption (1.0 vs. 3.1 days, *p* < 0.001 in RC; 0.8 vs. 2.6 days, *p* < 0.001 in LC) (Table 3). RAS patients also demonstrated significantly faster recovery milestones, including earlier IV fluid removal (5.1 vs. 9.3 days, *p* < 0.001 in RC; 4.1 vs. 7.4 days, *p* < 0.001 in LC) and shorter hospital stays (6.5 vs. 10.2 days in RC; 5.5 vs. 8.2 days in LC) (Table 3).

For complications in RC and LC patients after propensity score matching (PSM), RAS was associated with a lower rate of minor complications in LC (28.0% vs. 34.0%, *p* = 0.419 in RC; 17.1% vs. 27.1%, *p* = 0.028 in LC), including a significantly reduced risk of ileus (14.0% vs. 26.5%, *p* = 0.064 in RC; 6.2% vs. 15.9%, *p* = 0.007 in LC). Although rates of cardiovascular events, respiratory complications, urinary complications, and chyle leakage were lower in the RAS group, these differences were not statistically significant. The risk of major complications was similar between groups (4.0% vs. 7.0%, *p* = 0.746 in RC; 4.7% vs. 3.1%, *p* = 0.564 in LC), including rates of anastomotic leakage (0.0% vs. 2.5%, *p* = 0.586 in RC; 3.1% vs. 1.2%, *p* = 0.228 in LC). The readmission rate was similar between each group (8.0% vs. 7.0%, *p* = 0.764 in RC; 9.3% vs. 5.4%, *p* = 0.151), mainly due to minor complications.

### 3.5. Diuretic Phase

The postoperative fluid balance was closely monitored using the hospital’s electronic medical records, which tracked key parameters, such as intravenous (IV) fluid administration, urine output, and gastrointestinal intake. The calculation of fluids and liquid foods was combined and recorded as the total weight.

**Figure 2:**
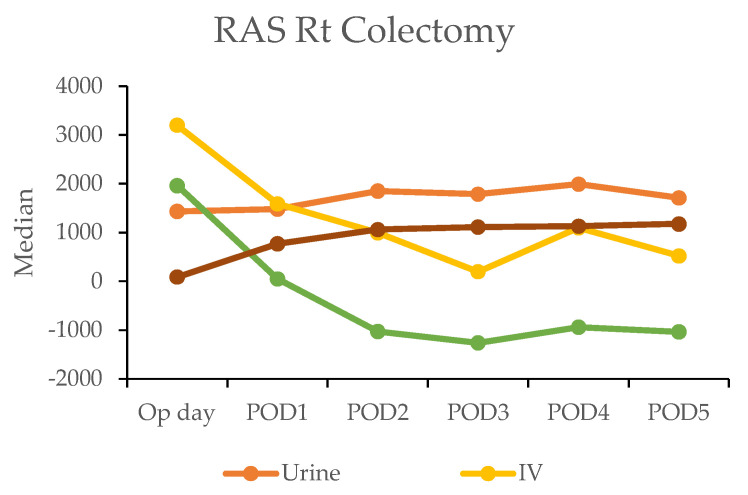

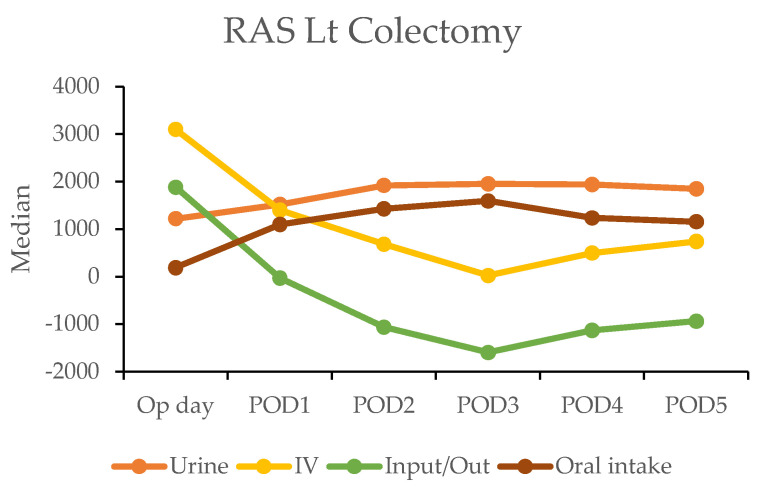
Daily urine output, intravenous fluid input, and oral intake in robotic-assisted surgery (RAS) patients by postoperative day (POD).

In the robotic-assisted surgery (RAS) group, the urine output remained stable over the subsequent days. The IV fluid amount was higher on the operation day due to the fluid given during the operation (Table 4). A notable negative fluid balance (IV fluid minus urine output (input/output)) was observed on postoperative day two for RC (−1025 mL) and day one for LC (−30 mL). This indicated the transition from the inflammatory to diuretic phases (Figure 2).

**Figure 3:**
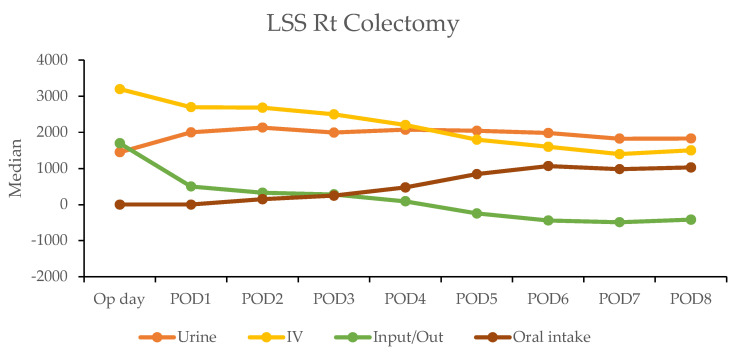

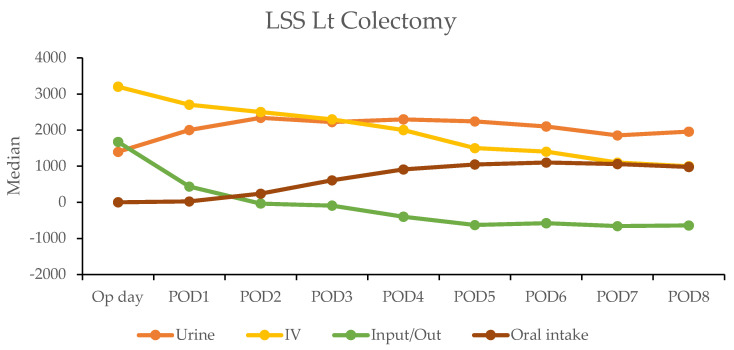
Daily urine output, intravenous fluid input, and oral intake in laparoscopic surgery (LSS) patients by postoperative day (POD).

In contrast, the laparoscopic surgery (LSS) group exhibited a more gradual recovery. A negative fluid balance was not achieved until postoperative day five in RC and day three in LC, indicating a delayed transition to the diuretic phase compared with the RAS group (Figure 3). This slower fluid management in LSS patients may have contributed to their longer recovery times. Additionally, gastrointestinal recovery was significantly faster in the RAS group, with patients achieving stable oral intake (>1000 mL per day) by postoperative day two; conversely, LSS patients did not reach this milestone until day six in RC and day five in LC (Table 4).

### 3.6. Pain Scores

**Figure 4:**
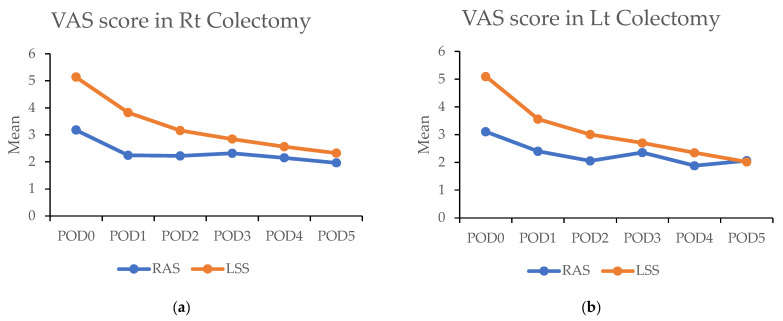

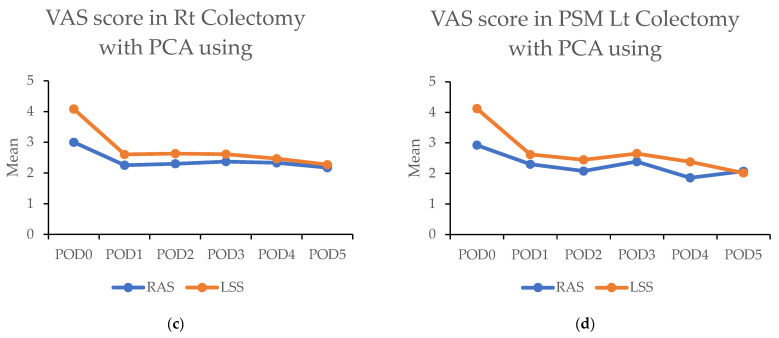
(**a**,**b**) Figures showing highest visual analog scale (VAS) pain scores by postoperative day (POD) in robotic-assisted surgery (RAS) and laparoscopic surgery (LSS). Patients underwent right colectomy and left colectomy. (**c**,**d**) Figures showing highest visual analog scale (VAS) pain scores by postoperative day (POD) in robotic-assisted surgery (RAS) and laparoscopic surgery (LSS) patients using patient-controlled analgesia (PCA).

In the propensity matched cohort, the daily maximum pain scores post surgery were consistently lower in the RAS group compared with the LSS group. On the day of surgery, the RAS group reported significantly lower pain scores than the LSS group in both RC [3.1 (±2.0) vs. 5.1 (±2.3); *p* < 0.001) and LC (3.1 (±1.7) vs. 5.1 (±2.4); *p* < 0.001). This difference persisted over the subsequent five days (Figure 4a,b and Table 5).

The significantly higher use of patient-controlled analgesia (PCA) in the RAS group (91.1% vs. 32.7%; *p* < 0.001) may have contributed to these findings. A survey was conducted among patients to address this using PCA. In this subset (43 RAS vs. 60 LSS in RC; 120 RAS vs. 92 LSS in LC), the RAS group still demonstrated significantly lower pain scores on the day of surgery in RC (3.0 (±1.8) vs. 4.1 (±2.3); *p* < 0.001) and during the first four days in LC. Overall, lower pain levels were observed in RAS patients compared with LSS patients when PCA was used (Figure 4c,d and Table 5).

## 4. Discussions

### 4.1. Study Findings

Our study demonstrated that robotic-assisted surgery (RAS) for colon cancer has several advantages over laparoscopic-assisted surgery (LSS). RAS results in a higher yield of lymph nodes and a lower conversion rate to open surgery. RAS also promotes faster recovery. Patients undergoing RAS experienced an earlier onset of diuresis and quicker return of gastrointestinal function. Specifically, the incidence of bloating was reduced, with less postoperative pain. However, the rate of major complications, such as anastomotic leakage, was similar between RAS and LSS. Despite longer operative times for RAS, the overall hospital stay was shorter due to its benefits in recovery speed and reduced complications.

### 4.2. More LN Harvested and Better Oncological Results

Lymph node dissection has both diagnostic and therapeutic significance [21,22]. It helps confirm cancer staging, and if cancer cells are found in the lymph nodes, a complete dissection allows for accurate pathological staging. If necessary, further systemic treatments can be administered, improving treatment outcomes. In cases where colorectal cancer metastasizes to the aortic lymph nodes or the surface of the superior mesenteric artery (SMA), higher-level dissection is required. Robotic-assisted surgery (RAS) offers better visualization and a more stable platform, enabling precise lymph node dissection along the curved surfaces of blood vessels. The robotic arms also have bipolar functionality, which reduces the risk of lymphatic leakage, such as a chyle leak. If a chyle leak occurs, patients may need to switch to a fat-free diet or fasting, significantly delaying gastrointestinal recovery. Our study shows that RAS achieves a higher lymph node yield and a significantly lower rate of chyle leak, demonstrating better cancer treatment outcomes with fewer surgical complications.

Additionally, the advantages of the RAS platform enable surgeons to perform other tasks, such as high-level lymph node dissection with low ligation after clearing the vessels, ensuring better blood circulation to the anastomotic site and reducing anastomotic leakage [23]. For earlier-stage lesions, RAS allows for highly selective vascular preservation during colectomy, helping to maintain bowel function. Moreover, in cases where lateral lymph node metastasis requires lateral lymph node dissection, RAS provides precise dissection in the narrow and complex pelvic lateral vessels, reducing surgical complications [24,25]. Other studies have also confirmed RAS’s superior performance in these aspects. Overall, these factors highlight the advantages of RAS in lymph node dissection.

### 4.3. Lower Inflammatory Response and Faster Recovery

Managing the patient’s inflammatory response is a critical aspect of postoperative recovery. A study published in the *British Journal of Surgery* (*BJS*) reported that patients who underwent RAS had significantly lower C-reactive protein (CRP) [26] levels on the first postoperative day than those who underwent LSS in colon cancer surgery. This finding suggests that RAS causes a lower surgical stress response. Our study supports these findings, as patients in the RAS group demonstrated significantly faster recovery of gastrointestinal function, allowing for earlier resumption of oral intake and reduced dependence on intravenous fluids, contributing to a significantly faster diuretic phase, which is a key indicator of postoperative recovery. Additionally, while differences in early oral intake attempts between RAS and LSS were influenced by enhanced recovery after surgery (ERAS) protocols, RAS patients experienced less abdominal bloating and faster recovery of gastrointestinal function. These factors highlight how RAS reduces physical stress on patients, facilitating a smoother recovery process compared with LSS.

### 4.4. Minor Pain and Faster Recovery

Robotic-assisted surgery (RAS) results in less postoperative pain than laparoscopic-assisted surgery (LSS) due to its greater stability. Although both procedures typically involve five similar incisions, RAS is a more stable operating platform and reduces tissue manipulation. The concentric movement of the trocars reduces strain on the patient’s incisions, exerts less traction on the skin, and is gentler on the abdominal tissues during the operation. Meanwhile, LSS, particularly in challenging angles, may cause more inadvertent tissue damage due to the manual handling of the instruments by the surgeon.

A recent study in the *Journal of Robotic Surgery* also found that RAS is associated with lower pain levels and reduced morphine use. Our study supports these findings, showing that RAS patients experienced significantly lower pain scores, lasting up to the fifth day post surgery. This pain reduction was evident even in patients who used patient-controlled analgesia (PCA), highlighting RAS’s clear advantage in minimizing pain.

Reduced pain correlates with decreased use of intraoperative anesthesia and postoperative morphine, which helps prevent nausea, a common side effect. With less pain, patients can mobilize sooner, reducing anxiety and promoting quicker gastrointestinal recovery. This aligns with the current enhanced recovery after surgery (ERAS) protocols. Among our patients, RAS patients achieved early ambulation, catheter removal, and intravenous line discontinuation within the recommended three days post surgery, indicating improved ERAS compliance and better recovery outcomes. This characteristic makes RAS particularly suitable for older patients, who may have a lower tolerance for surgical stress.

### 4.5. Comparison with Other Studies

Like findings from classic trials, such as those comparing LSS with open surgery, LSS allows for quicker recovery compared with open surgery. RAS takes these advantages a step further to achieve better outcomes. Meanwhile, the results from a study published in the *World Journal of Surgical Oncology* (*WJSO*) [27] showed that RAS outperformed LSS in colon surgery in several key areas: lower conversion rates to open surgery, higher lymph node yields, shorter hospital stays in both RC and LC, and lower ileus rates in RC. Our findings are consistent with this, showing that RAS provides a clear advantage over LSS in colon surgeries in terms of better surgical outcomes.

### 4.6. Potential RAS Applications

In addition to the advantages of robotic-assisted surgery (RAS) in standard resections for stage I–III colon cancer, RAS offers several other potential benefits over laparoscopic surgery (LSS), such as the following:

Intracorporeal anastomosis: Unlike extracorporeal anastomosis, this technique reduces the risk of minor vessel injury during exteriorization (which can affect blood circulation), decreases the risk of mesenteric torsion and bowel twisting, and allows for more precise length measurement with minimal dissection to achieve adequate margins. Furthermore, it facilitates smaller, more strategically placed extraction incisions. Using the robotic-assisted surgery (RAS) platform and its endo-wrist capabilities, the surgeon can perform purse-string sutures to secure the anvil on the EEA stapler and complete the intracorporeal anastomosis. This approach allows for the incision to be positioned in the lower abdomen, reducing postoperative pain, lowering the risk of incisional hernias, or even facilitating alternative extraction methods. For example, specimens can be extracted through natural orifices, such as the vaginal wall, as part of natural orifice transluminal endoscopic surgery (NOTES).

Locally advanced T4 tumors: Historically, laparoscopic surgery for T4 tumors has been associated with a higher conversion rate to open surgery. However, a study published in the *British Journal of Surgery* [28] demonstrated that RAS significantly reduces conversion rates (8.9% vs. 17.9%, *p* = 0.006) to LSS; hence, it shortens hospital stays and generates better five-year survival rates (56.2% vs. 43.4%; *p* = 0.007). Better surgery recovery can help patients complete more systemic chemotherapy and improve oncological outcomes. Another study involving multi-visceral pelvic resections [29] showed similar faster recovery times and improved cancer survival rates with RAS. RAS enhances postoperative recovery while also potentially improving oncological outcomes.

Stage IV colorectal cancer with liver metastasis: While such surgeries traditionally required open procedures, RAS now enables complete robotic resections, including liver resections [30], with lower complication rates (31.4% vs. 57.6%; *p* = 0.014) and shorter hospital stays (mean: 8.0 [2.2] vs. 10.7 [5.4] days; *p* < 0.001).

### 4.7. Drawbacks and Future Directions

This study demonstrated that RAS offers better recovery outcomes than LSS in colon cancer surgery. However, the findings were somewhat influenced by the concurrent promotion of ERAS protocols during the RAS case collection period. These protocols introduced several new clinical care practices, such as encouraging early postoperative oral intake, avoiding nasogastric and drainage tubes, and the widespread use of patient-controlled analgesia devices, which contributed to RAS’s superior recovery results compared with LSS. Many ERAS concepts have also been adopted in all LSS procedures, leading to improved outcomes. Conducting a prospective study comparing RAS and LSS outcomes under standardized ERAS protocols could provide a more objective evaluation of RAS’s advantages in colon cancer surgery.

## 5. Conclusions

This study demonstrated that robotic-assisted surgery (RAS) offers significant advantages over laparoscopic-assisted surgery (LSS) in colon cancer resection, particularly in faster postoperative recovery, higher lymph node yield (RC), fewer ileus complications (LC) and reduced postoperative pain. Although RAS involves longer operative times, it enhances precision and control, contributing to faster recovery milestones and improving surgical outcomes.

## Figures and Tables

**Table 1:** Demographics and pathologic characteristics of patients undergoing right colectomy before and after propensity score. Abbreviations: Rt: right; RAS: robotic-assisted surgery; LSS: laparoscopic surgery; BMI: body mass index; ASA: American Society of Anesthesiologists; ERAS: enhanced recovery after surgery; PCA: patient-controlled analgesia; RH: right hemicolectomy.

	Original Data Rt Colectomy		*p* Value	Matched Data Rt Colectomy		*p* Value
	RAS (*n* = 50)	LSS (*n* = 241)		RAS (*n* = 50)	LSS (*n* = 200)	
Age						
Mean	72.9 ± 12.3	67.7 ± 12.3	0.007 **	72.9 ± 12.3	69.8 ± 10.8	0.085
Median	73.9 (64.7–84.3)	67.3 (61.0–77.7)	0.007 **	73.9 (64.7–84.3)	68.5 (63.2–78.7)	0.061
Age group			0.298			0.565
≤40	0 (0.0%)	7 (2.9%)		0 (0.0%)	0 (0.0%)	
41–60	9 (18.0%)	49 (20.3%)		9 (18.0%)	32 (16.0%)	
61–80	27 (54.0%)	141 (58.5%)		27 (54.0%)	124 (62.0%)	
>80	14 (28.0%)	44 (18.3%)		14 (28.0%)	44 (22.0%)	
Gender			0.944			0.899
Female	26 (52.0%)	124 (51.5%)		26 (52.0%)	102 (51.0%)	
Male	24 (48.0%)	117 (48.5%)		24 (48.0%)	98 (49.0%)	
BMI	24.2 ± 3.9	24.0 ± 3.9	0.749	24.2 ± 3.9	23.8 ± 3.7	0.439
BMI group			0.842			0.946
<18	2 (4.0%)	13 (5.4%)		2 (4.0%)	10 (5.0%)	
18–25	30 (60.0%)	135 (56.0%)		30 (60.0%)	121 (60.5%)	
>25	18 (36.0%)	93 (38.6%)		18 (36.0%)	69 (34.5%)	
ASA			0.497			0.596
1	6 (12.0%)	19 (7.9%)		6 (12.0%)	16 (8.0%)	
2	25 (50.0%)	139 (57.7%)		25 (50.0%)	112 (56.0%)	
3	19 (38.0%)	83 (34.4%)		19 (38.0%)	72 (36.0%)	
Surgery			1.000			1.000
RH	50 (100.0%)	239 (99.2%)		50 (100.0%)	199 (99.5%)	
T-colectomy	0 (0.0%)	2 (0.8%)		0 (0.0%)	1 (0.5%)	
Clinical stage			0.518			0.408
0	3 (6.0%)	12 (5.0%)		3 (6.0%)	12 (6.0%)	
1	12 (24.0%)	60 (24.9%)		12 (24.0%)	52 (26.0%)	
2	12 (24.0%)	81 (33.6%)		12 (24.0%)	68 (34.0%)	
3	23 (46.0%)	88 (36.5%)		23 (46.0%)	68 (34.0%)	
Bowel preparation	47 (94.0%)	224 (92.9%)	1.000	47 (94.0%)	187 (93.5%)	1.000
PCA	43 (86.0%)	74 (30.7%)	<0.001 **	43 (86.0%)	60 (30.0%)	<0.001 **
ERAS	41 (82.0%)	22 (9.1%)	<0.001 **	41 (82.0%)	16 (8.0%)	<0.001 **
Surgeon experience						
3~5 yr	0 (0.0%)	71 (29.5%)	<0.001 **	0 (0.0%)	58 (29.0%)	<0.001 **
6~10 yr	9 (18.0%)	44 (18.3%)	0.966	9 (18.0%)	35 (17.5%)	0.934
>11 yr	41 (82.0%)	126 (52.3%)	<0.001 **	41 (82.0%)	107 (53.5%)	<0.001 **

Chi-square test or Independent *t*-test/Mann–Whitney U test, Median (IQR). ** *p* < 0.01.

**Table 2:** Demographics and pathologic characteristics of patients undergoing left colectomy before and after propensity score. Abbreviations: Lt: left; RAS: robotic-assisted surgery; LSS: laparoscopic surgery; BMI: body mass index; ASA: American Society of Anesthesiologists; ERAS: enhanced recovery after surgery; PCA: patient-controlled analgesia; LH: left hemicolectomy; AR: anterior resection.

	Original Data Lt Colectomy		*p* Value	Matched Data Lt Colectomy		*p* Value
	RAS (*n* = 129)	LSS (*n* = 356)		RAS (*n* = 129)	LSS (*n* = 258)	
Age						
Mean	71.9 ± 11.3	63.2 ± 13.3	<0.001 **	71.9 ± 11.3	68.1 ± 13.3	0.003 **
Median	73.1 (63.2–80.9)	62.8 (53.2–73.6)	<0.001 **	73.1 (63.2–80.9)	67.3 (61.3–76.7)	0.003 **
Age group			<0.001 **			0.127
≤40	0 (0.0%)	11 (3.1%)		0 (0.0%)	0 (0.0%)	
41–60	21 (16.3%)	139 (39.0%)		21 (16.3%)	52 (20.2%)	
61–80	75 (58.1%)	162 (45.5%)		75 (58.1%)	162 (62.8%)	
>80	33 (25.6%)	44 (12.4%)		33 (25.6%)	44 (17.1%)	
Gender			0.461			0.942
Female	56 (43.4%)	168 (47.2%)		56 (43.4%)	111 (43.0%)	
Male	73 (56.6%)	188 (52.8%)		73 (56.6%)	147 (57.0%)	
BMI	24.9 ± 4.1	24.5 ± 3.8	0.345	24.9 ± 4.1	24.2 ± 3.5	0.083
BMI group			0.432			0.582
<18	5 (3.9%)	7 (2.0%)		5 (3.9%)	7 (2.7%)	
18–25	70 (54.3%)	206 (57.9%)		70 (54.3%)	153 (59.3%)	
>25	54 (41.9%)	143 (40.2%)		54 (41.9%)	98 (38.0%)	
ASA			0.532			0.215
1	12 (9.3%)	46 (12.9%)		12 (9.3%)	32 (12.4%)	
2	81 (62.8%)	219 (61.5%)		81 (62.8%)	138 (53.5%)	
3	36 (27.9%)	91 (25.6%)		36 (27.9%)	88 (34.1%)	
Surgery			0.875			0.834
LH	17 (13.2%)	45 (12.6%)		17 (13.2%)	36 (14.0%)	
AR	112 (86.8%)	311 (87.4%)		112 (86.8%)	222 (86.0%)	
Clinical stage			0.381			0.478
0	3 (2.3%)	7 (2.0%)		3 (2.3%)	6 (2.3%)	
1	33 (25.8%)	110 (30.9%)		33 (25.8%)	78 (30.2%)	
2	39 (30.5%)	121 (34.0%)		39 (30.5%)	88 (34.1%)	
3	53 (41.4%)	118 (33.1%)		53 (41.4%)	86 (33.3%)	
Bowel preparation	122 (94.6%)	346 (97.2%)	0.166	122 (94.6%)	249 (96.5%)	0.367
PCA	120 (93.0%)	124 (34.8%)	<0.001 **	120 (93.0%)	92 (35.7%)	<0.001 **
ERAS	122 (94.6%)	56 (15.7%)	<0.001 **	122 (94.6%)	39 (15.1%)	<0.001 **
Surgeon experience						
3~5 yr	0 (0.0%)	93 (26.1%)	<0.001 **	0 (0.0%)	68 (26.4%)	<0.001 **
6~10 yr	14 (10.9%)	58 (16.3%)	0.137	14 (10.9%)	40 (15.5%)	0.213
>11 yr	115 (89.1%)	205 (57.6%)	<0.001 **	115 (89.1%)	150 (58.1%)	<0.001 **

Chi-square test or Independent *t*-test/Mann-Whitney U test, Median (IQR). ** *p* < 0.01.

**Table 3:** Perioperative outcomes of right and left colectomies after propensity score matching. Abbreviations: RAS: robotic-assisted surgery; LSS: laparoscopic surgery;.

	Matched Right Colectomy		*p* Value	Matched Left Colectomy		*p* Value
	RAS (*n* = 50)	LSS (*n* = 200)		RAS (*n* = 129)	LSS (*n* = 258)	
Operation time, minute (IQR)	310.5 (271.5–352.0)	190.5 (144.0–242.5)	<0.001 **	264.0 (229.0–309.0)	187.0 (149.0–249.0)	<0.001 **
Blood loss mean (SD)	55.9 ± 87.0	50.6 ± 59.6	0.611	51.8 ± 119.9	48.2 ± 115.7	0.774
Open rate	0 (0.0%)	6 (3.0%)	0.603	1 (0.8%)	8 (3.1%)	0.282
Drainage tube	12 (24.0%)	156 (78.0%)	<0.001 **	18 (14.0%)	190 (73.6%)	<0.001 **
Stoma creation	0 (0.0%)	1 (0.5%)	1.000	4 (3.1%)	11 (4.3%)	0.576
pCRM+	1 (2.0%)	3 (1.5%)	1.000	2 (1.6%)	1 (0.4%)	0.259
Distal margin	7.1 ± 4.2	6.8 ± 6.6	0.789	3.2 ± 2.4	3.6 ± 2.8	0.235
LN harvest	31.4 ± 13.7	26.8 ± 10.6	0.028 *	25.8 ± 10.7	23.9 ± 9.2	0.066
Water intake, (day)	0.7 ± 1.0	2.4 ± 1.7	<0.001 **	0.5 ± 0.7	2.0 ± 1.5	<0.001 **
Liquid diet intake, (day)	1.0 ± 0.9	3.1 ± 2.0	<0.001 **	0.8 ± 0.8	2.6 ± 1.7	<0.001 **
Defecation (day)	2.1 ± 1.5	4.1 ± 2.6	<0.001 **	1.8 ± 1.8	3.7 ± 2.5	<0.001 **
Foley removal (day)	2.6 ± 3.5	5.3 ± 6.7	<0.001 **	2.0 ± 2.7	4.3 ± 3.7	<0.001 **
Day to DC IV (day)	5.1 ± 5.2	9.3 ± 7.5	<0.001 **	4.1 ± 5.0	7.4 ± 4.6	<0.001 **
Day to discharge (day)	6.5 ± 5.1	10.2 ± 8.6	0.005 **	5.5 ± 4.8	8.2 ± 4.7	<0.001 **
Textbook outcomes	21 (42.0%)	20 (10.0%)	<0.001 **	69 (53.5%)	37 (14.3%)	<0.001 **
Any grade Complications	15 (30.0%)	82 (41.0%)	0.153	27 (20.9%)	77 (29.8%)	0.062
Minor complications (all)	14 (28.0%)	68 (34.0%)	0.419	22 (17.1%)	70 (27.1%)	0.028 *
Major complications (all)	2 (4.0%)	14 (7.0%)	0.746	6 (4.7%)	8 (3.1%)	0.564
Mortality	0 (0.0%)	3 (1.5%)	1.000	1 (0.8%)	1 (0.4%)	1.000
Ileus	7 (14.0%)	53 (26.5%)	0.064	8 (6.2%)	41 (15.9%)	0.007 **
Cardiovascular	0 (0.0%)	2 (1.0%)	1.000	0 (0.0%)	5 (1.9%)	0.174
Pneumonia	2 (4.0%)	14 (7.0%)	0.746	4 (3.1%)	11 (4.3%)	0.576
UTI	0 (0.0%)	5 (2.5%)	0.586	2 (1.6%)	7 (2.7%)	0.724
Chyle	0 (0.0%)	4 (2.0%)	0.587	0 (0.0%)	7 (2.7%)	0.101
Wound inf.	5 (10.0%)	11 (5.5%)	0.328	2 (1.6%)	7 (2.7%)	0.724
Leakage	0 (0.0%)	5 (2.5%)	0.586	4 (3.1%)	3 (1.2%)	0.228
30 d re-admission	4 (8.0%)	14 (7.0%)	0.764	12 (9.3%)	14 (5.4%)	0.151
Re-ad due minor	3 (75.0%)	13 (92.9%)		10 (83.3%)	12 (85.7%)	
Re-ad due major	1 (25.0%)	1 (7.1%)	0.405	2 (16.7%)	2 (14.3%)	1.000

Chi-square test/Fisher’s exact test or Independent *t*-test. * *p* < 0.05, ** *p* < 0.01.

**Table 4:** Depiction of urine, intravenous input fluid, and oral intake on each postoperation day (POD) between RAS and LSS patients. Abbreviations: Rt: right; Lt: left; RAS: robotic-assisted surgery; LSS: laparoscopic surgery; Op day: operation day; POD: postoperative day.

Median	RAS Rt Colectomy					LSS Rt Colectomy				
	*n*	Urine	IV	Input/Out	Oral Intake	*n*	Urine	IV	Input/Out	Oral Intake
Op day	50	1435	3200	1965	90	200	1428	3200	1680	0
POD1	50	1477.5	1592.5	52.5	775	200	2000	2700	500	0
POD2	50	1852.5	1000	−1025	1061	200	2125	2600	340	142.5
POD3	45	1790	200	−1260	1116	199	1970	2500	310	240
POD4	26	1995	1100	−935	1136	191	2075	2200	114	450
POD5	20	1712.5	525	−1035	1179.5	179	2040	1850	−150	788
POD6						158	1985	1600	−357.5	1038
POD7						113	1850	1354	−550	992
POD8						79	1830	1400	−490	1026
**Median**	**RAS Lt Colectomy**					**LSS Lt Colectomy**				
	***n***	**Urine**	**IV**	**Input/Out**	**Oral intake**	***n***	**Urine**	**IV**	**Input/Out**	**Oral intake**
Op day	129	1220	3100	1880	190	258	1302.5	3200	1785	0
POD1	129	1520	1400	−30	1095	258	1905	2646	490	25
POD2	128	1925	680	−1066.5	1430.5	258	2252.5	2500	56	210
POD3	116	1955	25	−1595	1594	255	2180	2300	−50	547
POD4	50	1940	500	−1130	1238	235	2250	2100	−310	838
POD5	28	1850	745	−940	1158.5	214	2180	1630	−595	1029
POD6						163	2080	1400	−450	1054
POD7						112	1850	1100	−655.5	1038
POD8						73	1930	1000	−820	960

**Table 5:** Above: The highest visual analog scale (VAS) pain scores for each postoperative day (POD) in robotic-assisted surgery (RAS) and laparoscopic surgery (LSS) patients in right colectomy (RC) and left colectomy (LC). Below: VAS of each day in RAS and LSS patients with PCA used in RC and LC. Abbreviations: PSM: propensity score matching; RAS: robotic-assisted surgery; LSS: laparoscopic surgery. * *p* < 0.05, ** *p* < 0.01.

VAS Score in PSM Rt Colectomy						VAS Score in PSM Lt Colectomy				
	*n*	RAS	*n*	LSS	*p* Value	*n*	RAS	*n*	LSS	*p* Value
POD0	50	3.2 ± 2.0	200	5.1 ± 2.3	<0.001 **	129	3.1 ± 1.7	258	5.1 ± 2.4	<0.001 **
POD1	50	2.2 ± 0.7	200	3.8 ± 1.9	<0.001 **	129	2.4 ± 1.4	258	3.6 ± 1.8	<0.001 **
POD2	50	2.2 ± 1.4	200	3.2 ± 1.7	<0.001 **	129	2.1 ± 1.2	258	3.0 ± 1.7	<0.001 **
POD3	50	2.3 ± 1.4	199	2.8 ± 1.7	0.011 *	129	2.4 ± 1.7	258	2.7 ± 1.6	0.001 **
POD4	45	2.2 ± 1.5	198	2.6 ± 1.6	0.041 *	110	1.9 ± 1.3	255	2.3 ± 1.5	0.001 **
POD5	27	2.0 ± 1.2	192	2.3 ± 1.4	0.292	47	2.1 ± 1.3	237	2.0 ± 1.3	0.936
	**VAS Score in PSM Rt Colectomy with PCA Using**					**VAS Score in PSM Lt Colectomy with PCA Using**				
	***n***	**RAS**	***n***	**LSS**	***p* Value**	***n***	**RAS**	***n***	**LSS**	***p* Value**
POD0	43	3.0 ± 1.8	60	4.1 ± 2.3	0.011 *	120	2.9 ± 1.6	92	4.1 ± 2.5	<0.001 **
POD1	43	2.3 ± 0.7	60	2.6 ± 1.3	0.141	120	2.3 ± 1.3	92	2.6 ± 1.2	0.028 *
POD2	43	2.3 ± 1.4	60	2.6 ± 1.5	0.076	120	2.1 ± 1.2	92	2.4 ± 1.4	0.039 *
POD3	43	2.4 ± 1.3	60	2.6 ± 1.7	0.569	120	2.4 ± 1.8	92	2.7 ± 1.7	0.083
POD4	39	2.3 ± 1.5	58	2.5 ± 1.8	0.843	102	1.9 ± 1.3	89	2.4 ± 1.5	0.012 *
POD5	23	2.2 ± 1.2	55	2.3 ± 1.5	0.957	44	2.1 ± 1.4	75	2.0 ± 1.1	0.757

## Data Availability

All data generated or analyzed during this study are included in this published article. The original datasets can be made available upon reasonable request to the corresponding author.
